# Electromyographic patterns and masticatory muscles activity changes in patients with dentoskeletal deformities before and after orthognathic surgery: a pilot study

**DOI:** 10.1007/s10006-026-01567-z

**Published:** 2026-05-13

**Authors:** Stefania Troise, Federica Calabria, Vitanna Canterino, Umberto Committeri, Gabriele Monarchi, Giuseppe Consorti, Giovanni Salzano, Gianluca Renato De Fazio, Marco Friscia, Paola Bonavolontà, Vincenzo Abbate, Giovanni Dell’Aversana Orabona

**Affiliations:** 1 Maxillofacial Surgery Unit, Department of Neurosciences, Reproductive and Odontostomatological Sciences, University of Naples Federico II, Naples, Italy; 2 Maxillo-Facial Surgery Unit, Azienda Ospedaliera Santa Maria di Terni, Terni, Italy; 3 Department of Medicine, Section of Maxillo-facial surgery, University of Siena, Siena, Italy; 4 Division of Maxillofacial Surgery, Department of Neurological Sciences, Marche University Hospital-Umberto I, Ancona, Italy

**Keywords:** Surface electromyography, Orthognathic surgery, Dentoskeletal deformities, Occlusal contacts, TeethanTM

## Abstract

**Purpose:**

Orthognathic surgery involves repositioning the skeletal bases to correct dentoskeletal deformities and malocclusions. While its impact on occlusion is well documented, its effects on masticatory muscle activity remain under investigation. This pilot study evaluates changes in static electromyographic (sEMG) patterns of masseter and temporal muscles, and explores correlations with TMJ-related muscular symptoms before and after surgery.

**Methods:**

A surface electromyography (sEMG), by employing TeethanTM, based on occlusal contacts, was performed recording the activation patterns of masseter and temporal muscles, in ten patients with dentoskeletal malocclusions, undergoing orthognathic surgery. The registration assessed the muscles patterns sEMG preoperatively (T0), postoperatively at one month (T1), and at six months (T2) after surgery.

**Results:**

Across the cohort, temporal POC increased by 6.76 ± 8.36 from T0 to T1 and by 5.68 ± 5.33 from T1 to T2. Masseter POC rose by 8.74 ± 7.38 (T0–T1) and 8.12 ± 7.42 (T1–T2). Barycentre values shifted by 7.36 ± 4.13 (T0–T1) and 10.65 ± 9.17 (T1–T2), indicating progressive rebalancing of occlusal force distribution. Patients with Class III malocclusion showed greater masseter activation post-surgery, while Class II patients exhibited increased temporal muscle activity. Three patients with preoperative muscular discomfort reported complete symptom resolution at T2, while one patient experienced mild residual symptoms.

**Conclusion:**

Data obtained from this preliminary study seem to confirm that skeletal bases repositioning modifies the occlusal contacts and, consequently, the neuromuscular proprioceptive stimuli and the activation of the masticatory muscles. sEMG may provide useful complementary information on neuromuscular adaptation following orthognathic surgery. However, larger controlled studies with standardized outcomes and statistical analyses are required before drawing definitive conclusions or recommending its routine clinical use.

## Introduction

Orthognathic surgery (OS) is a well-known procedure for treating malocclusions and dentoskeletal deformities, with the goal of enhancing masticatory function and facial aesthetics. However, the impact of these surgical procedures on the masticatory muscular system, in terms of changes in muscle activation patterns and proprioception, is still being studied. Proprioception of the masticatory muscles plays a crucial role in maintaining neuromuscular balance and post-operative functional adaptation. Alteration of occlusal contacts through orthognathic surgery can modify these proprioceptive inputs, temporarily or permanently affecting neuromuscular control and motor co-ordination [1].

Changes in masticatory function can be recorded by means of surface electromyography (sEMG) [2]. One of the main tools to objectify changes in electromyographic patterns after orthognathic surgery is surface elettromyographhy (sEMG). It provides information on neuromuscular alterations induced by occlusal contact [3]. When the contact points tend to concentrate on molars, the masseters record a greater contraction than the corresponding temporal muscles (rear barycenter). Conversely, in the occlusal condition where the barycentre moves to the antero-lateral sectors, the temporal muscles state greater contractile forces (front barycentre) [4].

Masticatory forces are modulated by occlusal input proprioception, based on activation of periodontal receptors [5]. Proprioception is essential for neuromuscular balance and post-operative functional adaptation, and changes in occlusal contacts following surgery can influence proprioceptive inputs, potentially affecting motor coordination and neuromuscular control [6].

Recent reviews were indicating that dentofacial deformities could be a risk factor for occurrence of temporomandibular disorders (TMD), including arthralgia, disc displacement, joint clicking, myofascial pain, deviation on opening, grinding, headache, joint crepitation, muscle palpation tenderness and pain on palpation of the temporomandibular joint (TMJ) [7, 8]. Combining orthodontic and surgical treatments for malocclusions has been shown to affect TMJ health. However, publications regarding the risk factors that predict negative TMJ outcomes after orthognathic surgery are scarce. The bibliometric analysis conducted by Grillo et al. [9] revealed an increasing interest in research on this subject, demonstrated by a growing number of English-language publications with high citation counts. The study emphasized the necessity of comprehensive evaluation, treatment, and follow-up for TMD in individuals who have undergone OS, while also highlighting the need for more research and an unified approach to management strategies. Importantly, patients did not undergo OS specifically because of TMD, making it crucial to monitor the progression of TMD in these cases.

Several systematic reviews and meta-analyses have underscored the shortage of properly designed clinical trials. For instance, in 2009, Al-Riyami et al. [10] conducted a systematic review and concluded that while orthognathic surgery should not be performed solely to treat TMD, patients undergoing combined orthodontic and surgical treatment who also have TMD may be more likely to experience improvement in their symptoms rather than worsening. In 2013, Chauvel-Lebret et al. [11] reviewed the topic and found that surgery had varying and unpredictable impacts on TMD.

In 2017, Al-Moraissi et al. [12] conducted a systematic review and meta-analysis that included 29 studies and 5029 patients, with follow-up periods ranging from 4 months to 6.3 years. They discovered that orthognathic surgery alleviated TMD symptoms in many patients who had preoperative symptoms, while it induced symptoms in a smaller subset of patients who were asymptomatic before the procedure. The improvement, lack of change, or worsening of TMD symptoms was not influenced by the presence of pre-surgical symptoms or the type of skeletal deformity.

Finally, the most recent systematic review, carried out by Chenxinzi et al. [13] in 2025, evaluated 21 articles and concluded that OS may influence TMD symptoms in patients with dentofacial deformities, but due to its unpredictable outcomes and lack of a clear causal link, it should not be used as an indicator for TMD treatment, highlighting the need for thorough preoperative evaluation and further research into contributing factors.

This observatory study would evaluate the changes in static electromyographic patterns of masseter and temporal muscles before and after orthognathic surgery in patients with dentoskeletal deformities, contributing to a better understanding of the functional implications and post-operative stability in orthognathic surgery and in additional evaluated how and if muscular changes can impact in TMD.

The clinical relevance of this study is based on the possibility of routinely performing sEMG before planning and surgery and, therefore, using as a support tool in the management of patients with dento-skeletal deformities in order to comprehend how masticatory vectors can influence new habits contributing to a better understanding of the functional implications and post-operative remodelling in orthognathic surgery.

## Materials and methods

### Study design

A prospective observational pilot study was conducted at Maxillofacial Surgery Unit of the University of Naples Federico II between December 2024 and September 2025, on 10 patients with diagnosis of dentoskeletal malocclusion. This study was reviewed and approved by the Ethics Committee of the University of Naples Federico II, under protocol number CE 148/23.

Patients satisfied these inclusion criteria were enrolled in the study:

- Age > 18 years.
- Class II or III malocclusion according Angle classification [14].
- Orthognathic surgery, based on LeFort 1 osteotomy and Bilateral Sagittal Split Osteotomy (BSSO).
- Presurgical Orthodontics treatment.
- Consent to undergo surface electromyography (sEMG).
- Minimum follow-up 6 months.
- No diagnoses of TMJD following the Diagnostic Criteria for Temporomandibular Disorders (DC/TMD).

Patients who did not satisfy these inclusion criteria or met these exclusion criteria were not included in the study:

- Surgery first or surgery early;
- Performing only LeFort 1 osteotomy or only BSSO;
- Refusal to undergo sEMG;
- Unavailability of surface electromyography.
- TMJD diagnosticated following the Diagnostic Criteria for Temporomandibular Disorders (DC/TMD).

The sEMG registration was performed by TeethanTM (Teethan S.p.A., Italy) device.

### Surface electromyography (sEMG) registration

All the patients underwent the same protocol, performed using Teethan device.

TeethanTM device is a wireless medical tool designed for performing a sEMG based on the dental occlusion. sEMG is a non-invasive diagnostic technique used to measure the electrical activity produced by skeletal muscles. Тhe purpose of the sEMG study on masticatory muscles is to determine if there is any muscle imbalance due to occlusal interference. The tested areas are masseter and anterior temporalis muscles on both sides. The TeethanTM device consists of four wireless EMG probes, a USB receiver, a charging station, and pre-gelled electrodes.

The recording is performed by placing the probes on the skin overlying the muscles of interest to detect the electrical signals generated during muscle contractions. To standardize the sEMG recordings, all subjects are asked to maintain a comfortable posture, relax their arms by their sides, look straight ahead, and make no head or body movements during the test. All the measurements are conducted by the same two operators all the time, in order to minimize bias. Patients practice a maximum of two clenching tests, to prevent the muscle fatigue arising from their activity from altering the results: of them, the first is for calibration and then the test, both lasting 5 seconds.

Through the analysis, key evaluation indexes are obtained:

- POC (Position of the Occlusal Centre).
- BAR (Barycentre Assessment).
- TORS (Torsion Assessment).
- IMPACT (Impact Assessment).
- ASIM (Asymmetry Assessment).

The parameters evaluated by each index are described in Table 1.

**Table 1:** Electromyographic indices

Percent Overlapping Coefficient (POC)	assesses muscle contraction symmetry in both temporal and masseter pairs, showing balance between right and left sides. A value close to 100% indicates symmetrical muscle activity, while values below 83% suggest imbalance, influenced by dental contact affecting neuromuscular balance.
Barycentre (BAR)	assesses the position of the occlusal barycentre by comparing the contraction between temporals and masseters muscles. Greater masseter contraction is seen when contact points are on molars, while greater temporal force occurs when the barycentre shifts to the front. A BAR value above 90% indicates normal function.
Torsion (TORS):	measures the torsion attitude of the mandible in the horizontal plane. A normal value (> 90%) indicates no lateral displacement. Values below 90% suggest muscle pair dominance, causing jaw deviation to the right or left. “R” indicates right torsion, “L” indicates left torsion.
Impact (IMP):	measures muscle activity related to bite force. Normal values are between 100% and 115%. Higher values may indicate clenching, while lower values suggest acute proprioceptive inhibition or chronic pain.
Asymmetry (ASIM)	compares the activity of the right and left muscles. A positive value indicates greater activation on the right, while a negative value indicates greater activation on the left. Normal values range from − 10 to 10.
Global Index of Neuromuscular Balance	represented with a color-coded biofeedback ring: Green: when the global balance is greater than 83%. Yellow: when the global balance is between 82% and 75% Red: when the global balance is less than 74%.

### Temporomandibular disorders (TMD) assessment

All patients underwent a preoperative clinical screening for temporomandibular disorders (TMD) based on the Diagnostic Criteria for Temporomandibular Disorders (DC/TMD). The evaluation included palpation of the masticatory muscles and temporomandibular joints, assessment of mandibular deviation, maximum mouth opening, joint sounds (clicks), and functional history. None of the patients presented with signs of joint damage or disc displacement. However, four patients reported mild muscular pain during palpation of the masseter or temporalis muscles. These cases were not treated as a separate subgroup but were monitored within the overall cohort to explore potential correlations between skeletal repositioning, occlusal rebalancing, and neuromuscular adaptation. This approach reflects current recommendations in the literature, which advocate for integrated functional assessment in orthognathic patients, even in the absence of overt joint dysfunction. sEMG was used as a complementary tool to monitor muscle activity patterns potentially associated with muscular discomfort. This protocol was informed by recent literature emphasizing the role of occlusal proprioception and neuromuscular balance in the functional adaptation of the masticatory system following orthognathic surgery.

### Data collection and study protocol

Data on age, sex, type of malocclusion and dento-skeletal deformity, Virtual Surgical Planning, and type of surgery were collected for all patients. A sEMG was recorded for each patient at three different times:

- Preoperatively, the day before the surgery (T0).
- postoperatively, one months after surgery (T1), time until which patients wear orthodontic elastics.
- postoperatively, at six months after surgery (T2).

For all ten patients the same parameters were, therefore, recorded.

Below, we present a Class III malocclusion example case and a Class II malocclusion example case.

### Case 1: class III malocclusion

**Fig. 1:**
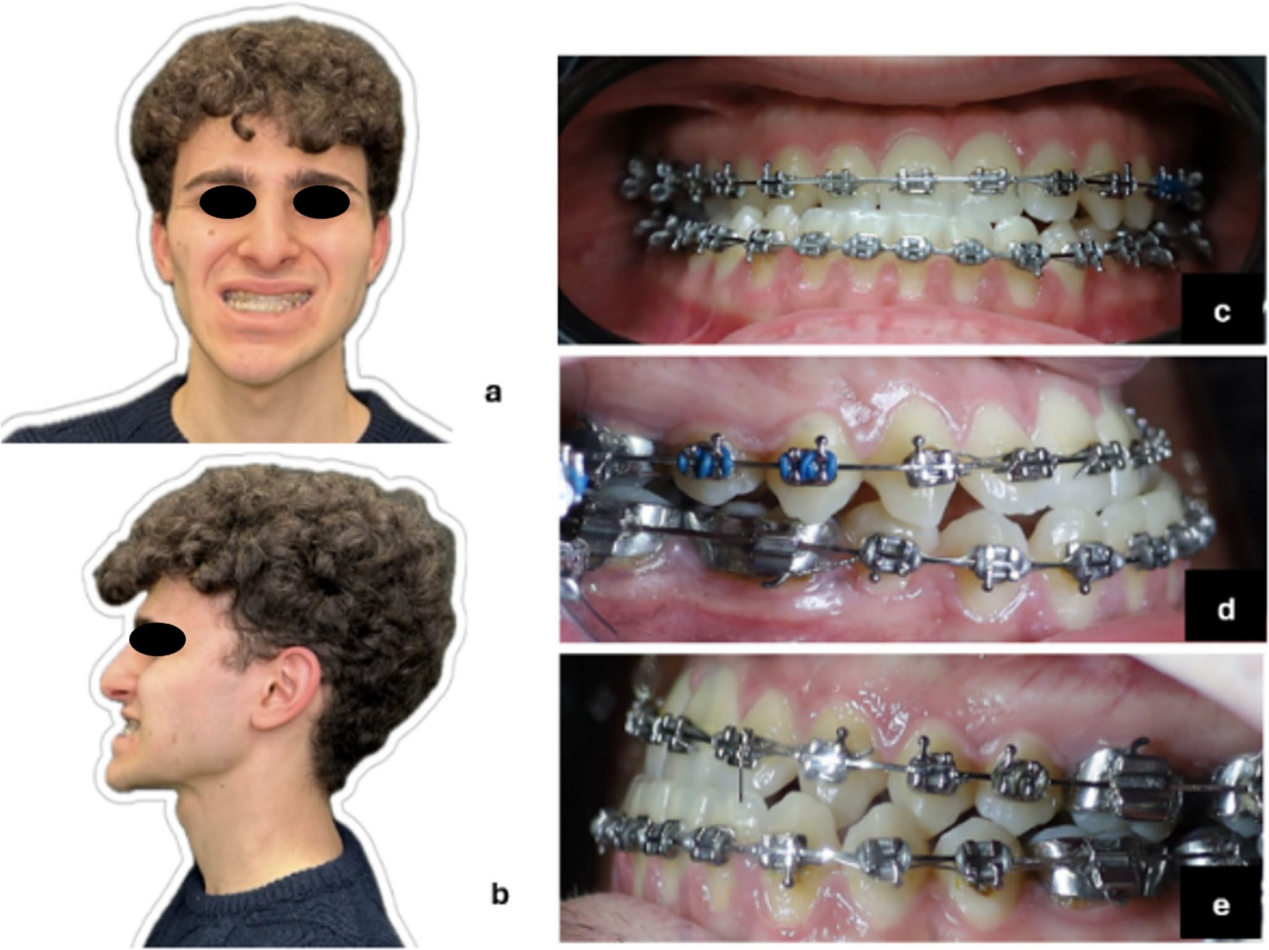
Preoperative images of case 1: (**a**) frontal view (**b**) lateral view (**c**) frontal occlusion (**d**) right lateral occlusion (**e**) left lateral occlusion

The patient P-1 was an 18-year-old Caucasian presenting with Class III dentoskeletal malocclusion. Clinical examination reveals a long facial morphology with mandibular latero-deviation to the right. Intraoral findings include a right-sided crossbite, left-sided scissor bite, and a negative overjet with a deviation of the interincisal line to the right, as shown in Fig. 1.

Although no joint pathology was detected, the pronounced asymmetry and altered occlusal load suggest a muscular imbalance compatible with TMD of myogenic origin. This is supported by the sEMG findings at T0, which showed elevated ASIM values and a lateralized POC pattern.

**Fig. 2:**
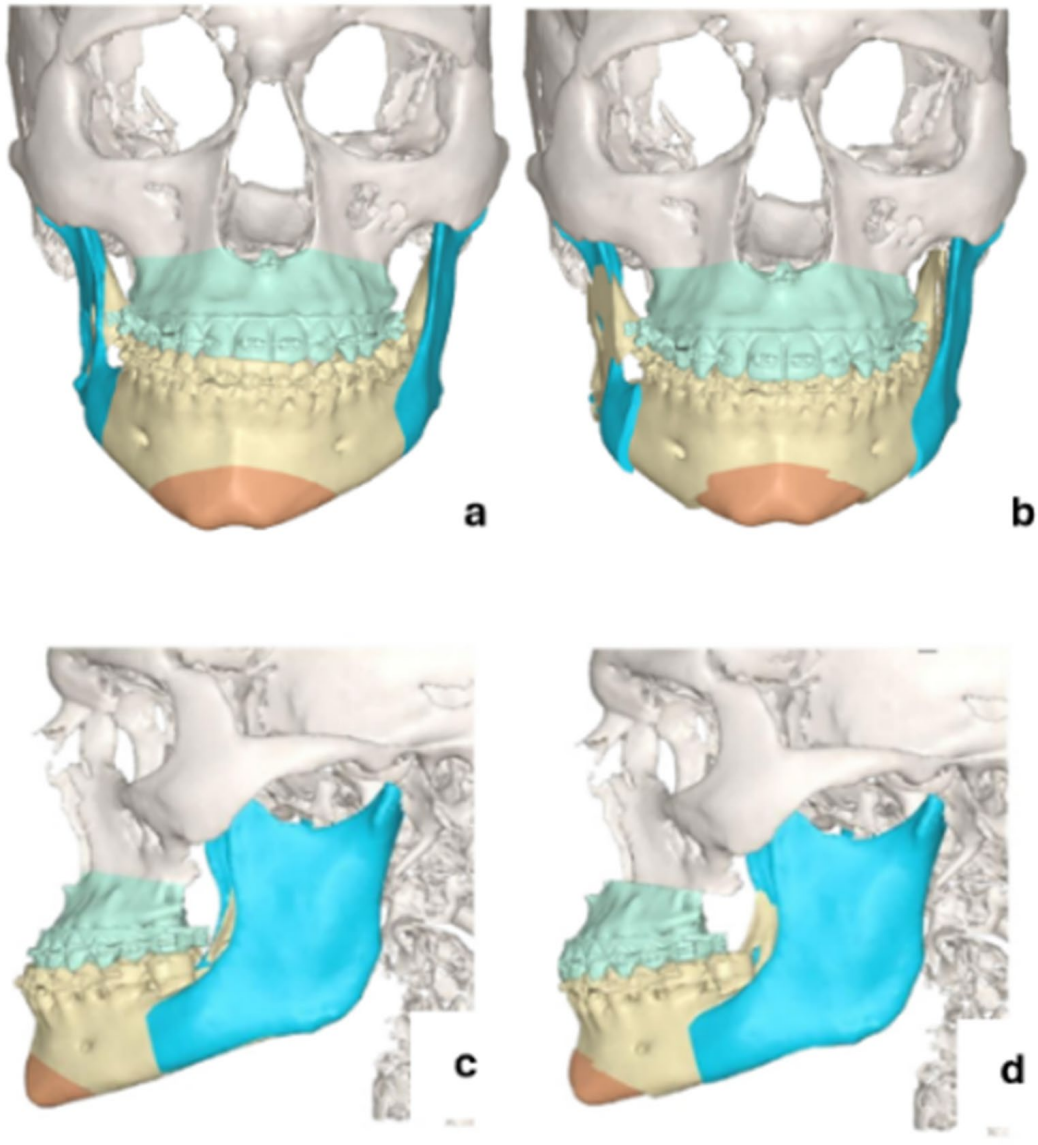
Virtual surgical planning of case 1 (**a**) preoperative frontal view (**b**) post-operative frontal view (**c**) pre-operative left lateral view (**d**) post-operative left lateral view

**Fig. 3:**
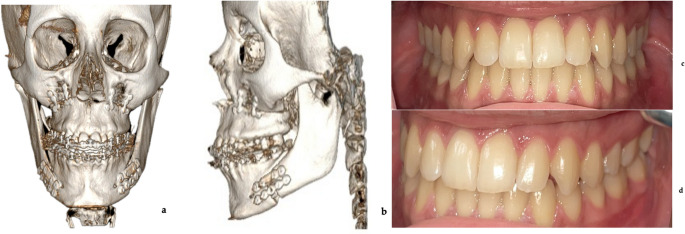
Post-operative images of case 1 (**a**) post-operative frontal view CT scan (**b**) post-operative lateral view CT scan (**c**) final occlusion in frontal view (**d**) final left lateral occlusion

The patient underwent bimaxillary repositioning surgery, including a maxillary advancement of approximately 5 mm, and bilateral mandibular osteotomies with a leftward lateralization of approximately 3 mm, as virtually planned (Fig. 2). The postoperative CT scan is shown in Fig. 3.

**Fig. 4:**
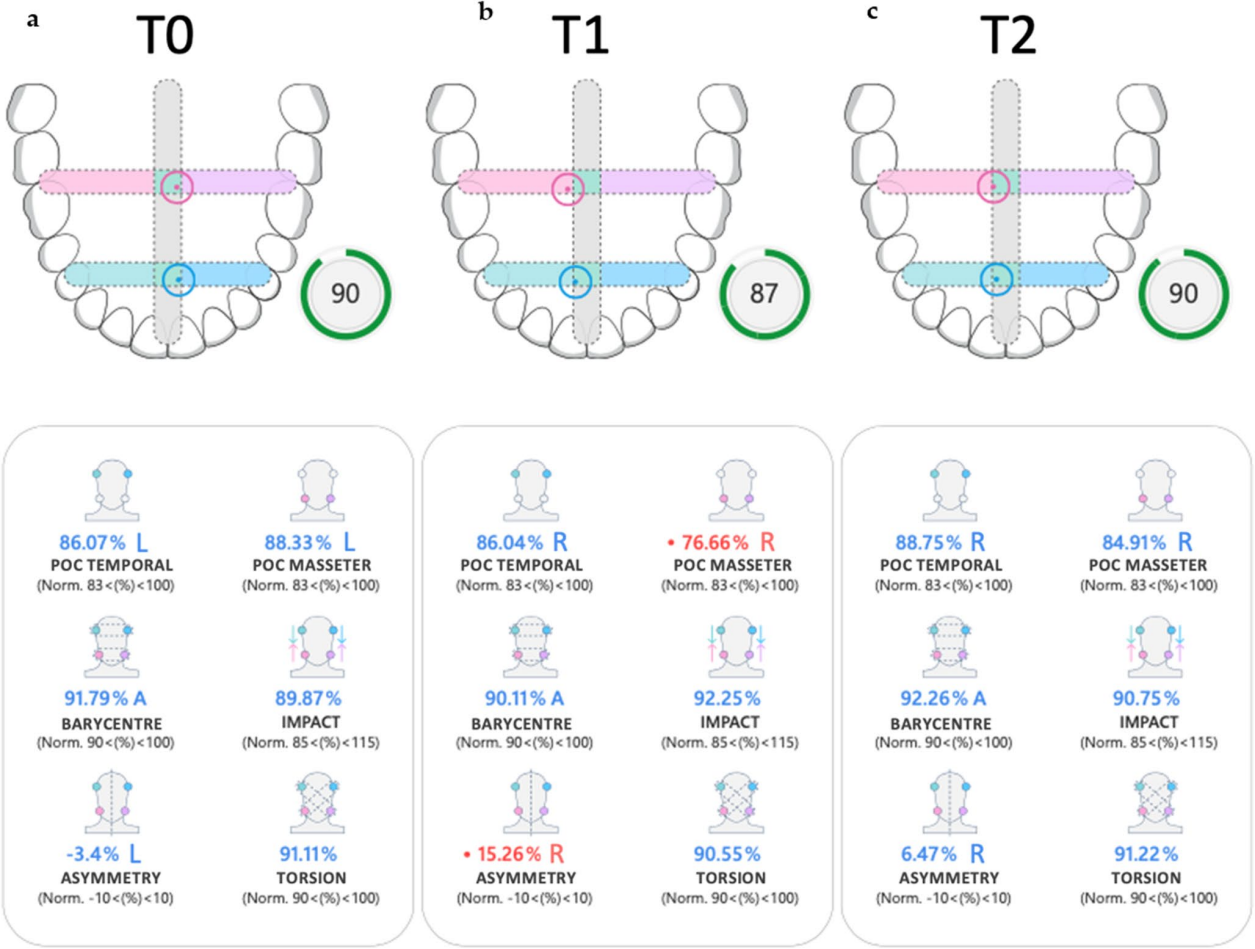
EMG patterns of Case 1 (**a**) EMG in T0 (**b**) EMG in T1 (**c**) EMG in T2. R: right side L: Left side; A: anterior

TeethanTM recordings of the patient at time T0, T1 and T2 are shown in Fig. 4;

preoperatively (T0), the patient exhibited pronounced asymmetry in muscle activation, with dominant left-sided activity in both masseter and temporalis muscles. The barycentre was anteriorized, and the ASIM index exceeded the physiological range (–13), indicating significant lateral imbalance. The IMPACT value was reduced (91%), suggesting diminished bite force and possible proprioceptive inhibition. Clinically, the patient reported mild discomfort in the left masseter region, consistent with muscular TMD features, although no joint pathology was present.

At one month postoperatively (T1), sEMG revealed a shift in dominance toward the right masseter, with ASIM values reversing direction (+ 11), reflecting neuromuscular instability during the early adaptation phase. IMPACT remained suboptimal (94%), and the patient continued to report mild muscular tension, particularly during clenching. These findings are consistent with the transitional phase of neuromuscular reorganization following skeletal repositioning.

By six months (T2), the patient demonstrated improved bilateral muscle coordination, with ASIM values returning within normal range (–4) and IMPACT increasing to 106%, indicating restored bite force and functional balance. However, the patient continued to report occasional mild discomfort during prolonged mastication, suggesting residual muscular sensitivity despite overall neuromuscular normalization.

###  Case 2: class II malocclusion 

**Fig. 5:**
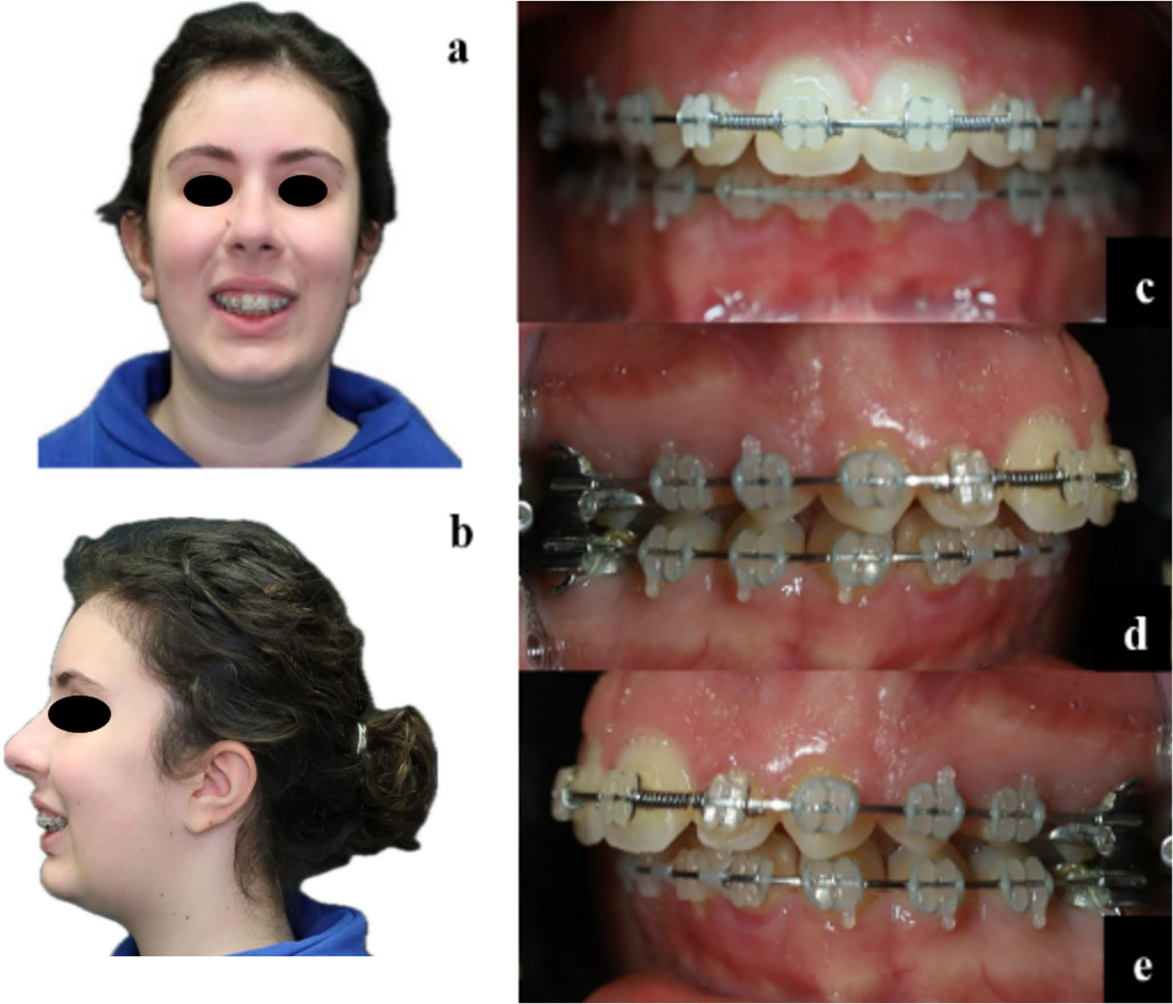
Preoperative images (**a**) Preoperative patient’s frontal view (**b**) Preoperative patient’s lateral view (**c**) frontal occlusion in II Angle’s class (**d**) right lateral occlusion (**e**) left lateral occlusion;

P-2 was an 18-year-old Caucasian patient presents with Class II dentoskeletal malocclusion. Physical examination reveals a short face with hypoplasia of the lower third of the face. Intraoral examination shows an overjet of 4 mm and the presence of an overbite (Fig. 5).

Preoperatively, the patient reported mild discomfort localized to the masseter muscles, without any signs of joint pathology or disc displacement. This symptomatology was consistent with muscular TMD, likely secondary to occlusal imbalance and compensatory hyperactivity.

**Fig. 6:**
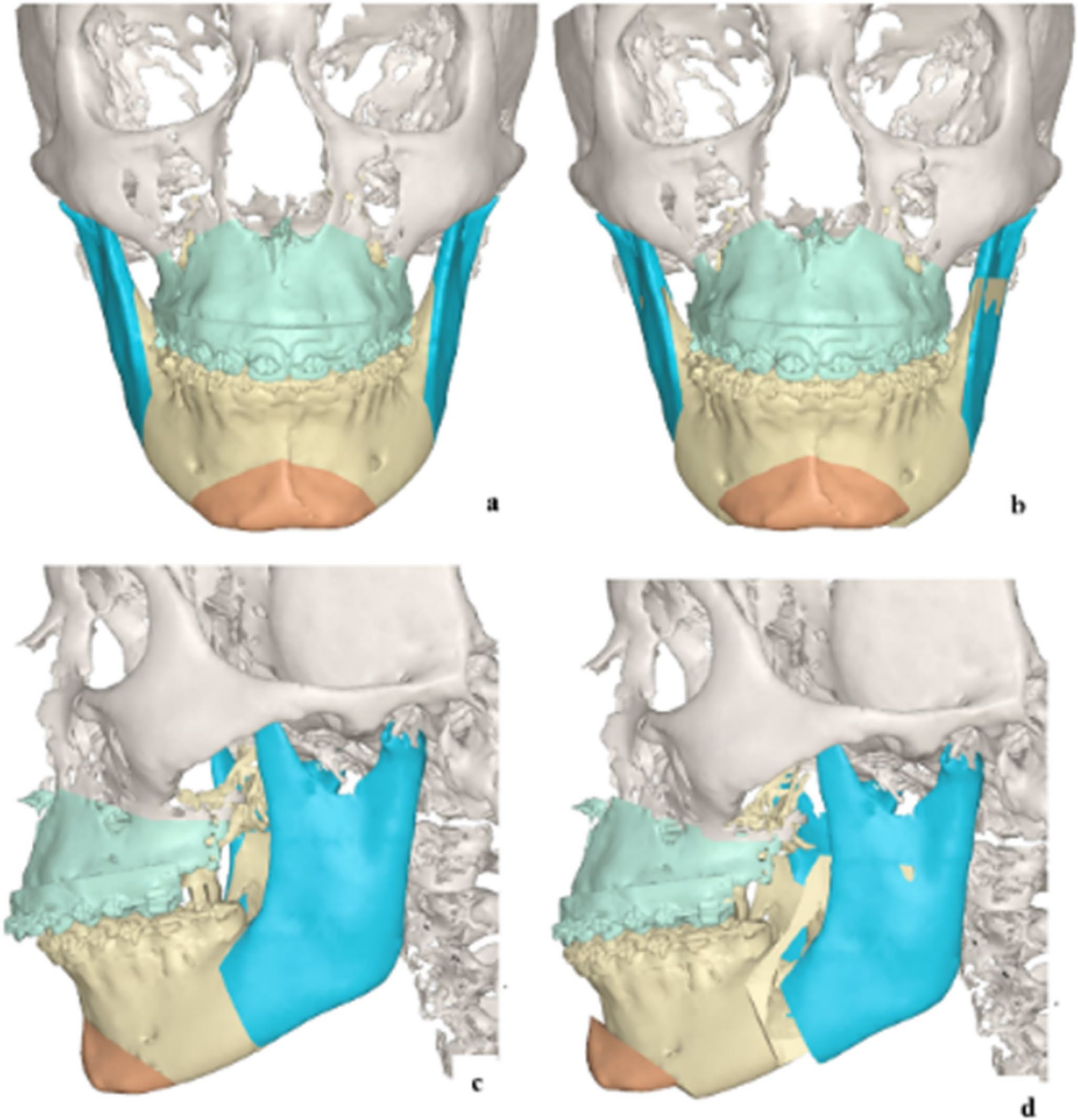
Virtual surgical planning. (**a**) preoperative frontal view (**b**) post-operative frontal view: maxillary advancement, bilateral BSSO, genioplasty (**c**) pre-operative lateral view (**d**) post-operative lateral view

**Fig. 7:**
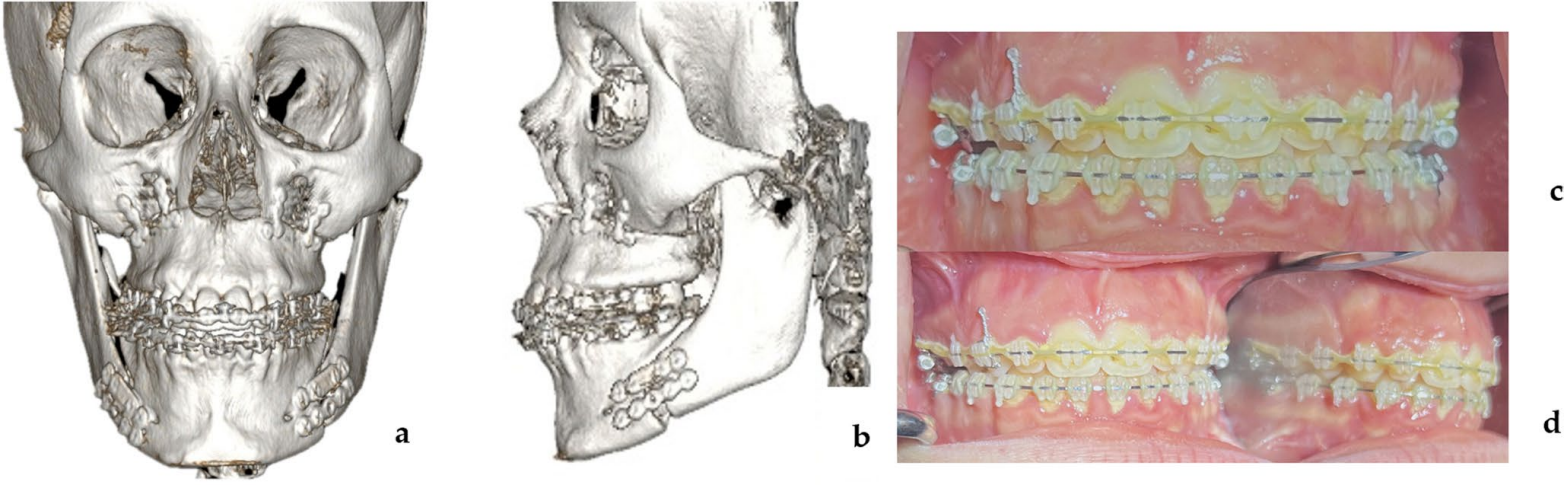
Post-operative images of Case 2 (**a**) post-operative frontal view CT scan (**b**) post-operative lateral view CT scan (**c**) final occlusion in frontal view (**d**) final left lateral occlusion

The patient underwent bimaxillary repositioning surgery with a maxillary advancement of approximately 2.75 mm and a mandibular advancement of approximately 8 mm, as virtually planned (Fig. 6). Post-operative CT scan is shown in Fig. 7.

**Fig. 8:**
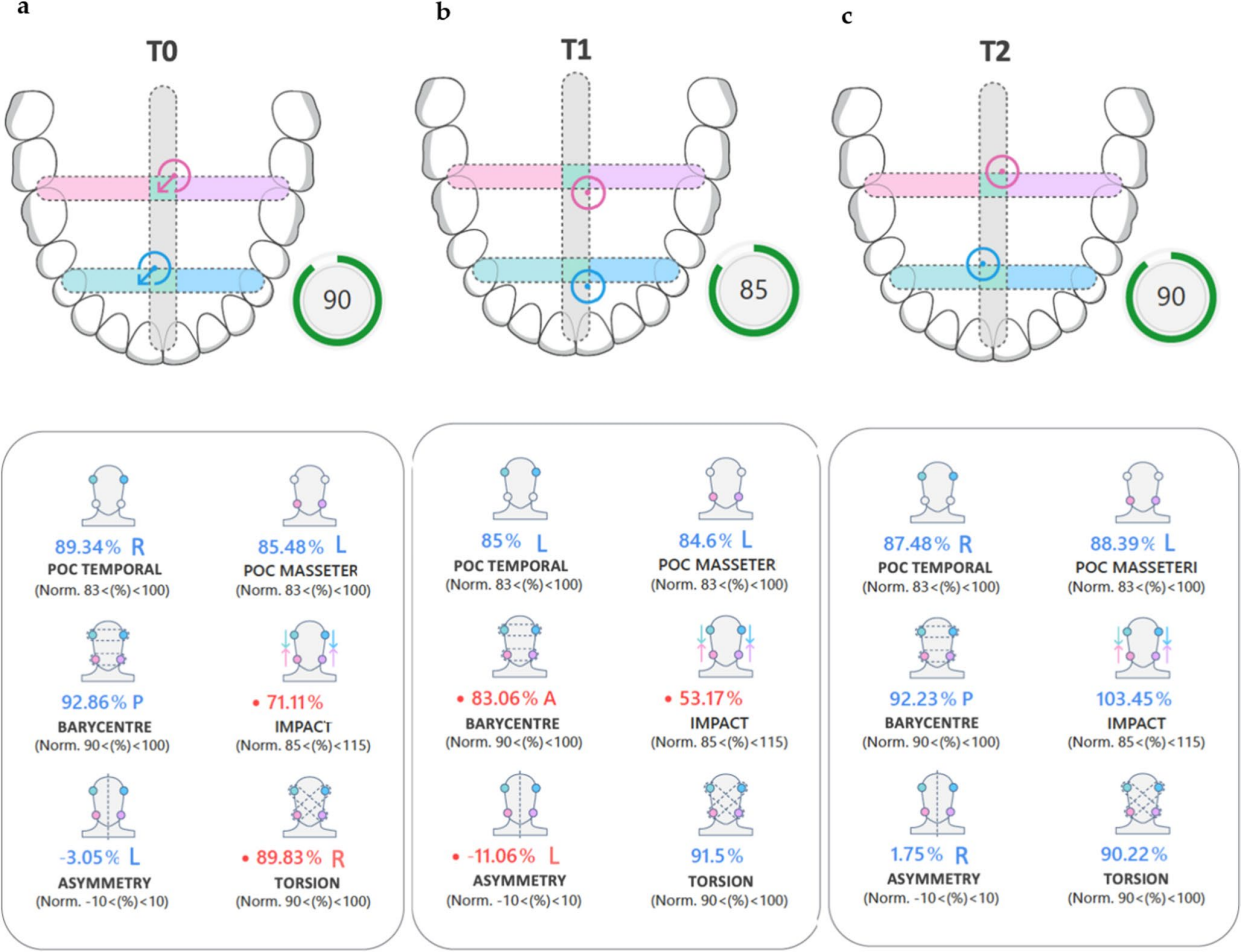
EMG patterns of case 2 (**a**) EMG in T0 (**b**) EMG in T1 (**c**) EMG in T2. R: right side L: Left side; A: anterior

The patient’s TeethanTM recordings at time T0, T1 and T2 are shown in Fig. 8.

sEMG in T0 shows a posterior barycentre, elevated asymmetry index (ASIM), a low impact due to major activation of masseter muscle, with a right torsional muscle activity, with a prevalence of right temporal muscle in POC and predominance of left masseter;

sEMG in T1 showed a transient anteriorization of the barycentre and persistent left-sided asymmetry, reflecting the neuromuscular adaptation phase following skeletal repositioning. The global index was reduced, consistent with temporary musculoskeletal imbalance during early healing and orthodontic elastic wear.

By six months (T2), the patient demonstrated normalization of sEMG parameters, including improved POC values, balanced barycentre, and restored IMPACT scores. Clinically, the muscular discomfort had resolved, and the occlusion was stable in Class I.

## Results

### Population

This is intended to be a pilot study hence a limited number of patients were enrolled: 3 female patients and 7 male patients, whom had a class II dentoskeletal deformities in 4 cases and a class III dentoskeletal deformities in 6 cases. The median age was 23.9 ± 4.1 years.

Population features, such us age, sex, defect, clinical examination and surgical planning were summarized in Table 2.

**Table 2:** Patients’ clinical and surgical features

Case	Age	Sex	Type of malocclusion	Physical Examination features	Surgery and movements
P-1	18	M	Class III dentoskeletal	long face, right later-deviation of 5 mm	Le Fort I: maxillary advancement 5 mm BSSO: mandibular advancement 3 mm
P-2	18	F	Class II dentoskeletal II division	Short face, overjet 4 mm, overbite 2 mm	Le Fort I: maxillary advancement 2.75 mm BSSO: mandibular advancement 8 mm
P-3	20	F	Class III dentoskeletal	long face, left later-deviation of 3.9 mm	Le Fort I: maxillary advancement 5.7 mm BSSO: mandibular left rotation 1.1°
P-4	38	M	Class II dentoskeletal II division	Short face, overjet 6 mm, overbite 3 mm	Le Fort I: maxillary advancement 2.75 mm BSSO: mandibular advancement 8 mm
P-5	29	M	Class III dentoskeletal	Long face, overbite 1 mm, overjet 2.2 mm	Le Fort I: maxillary advancement 5 mm and maxillary intrusion 1.5 mm BSSO: mandibular left rotation 2°
P-6	27	M	Class III dentoskeletal	Long face, open bite 0.64 mm	Le Fort I: maxillary advancement 6.7 BSSO: mandibular right rotation 1°
P-7	24	F	Class III dentoskeletal	Long face, left lateral deviation of 3.9 mm	Le Fort I: maxillary advancement of 5.7 mm, intrusion of 0.9 mm BBSO: mandibular right rotation of 1.1°
P-8	22	M	Class II dentoskeletal	Short face, biretrusive, overbite of 5.1 mm, overjet of 13.86 mm	Le Fort I: maxillary advancement 3 mm, intrusion 1 mm, counterclockwise 2.9° BSSO: mandibular right rotation 5.2°
P-9	21	F	Class II dentoskeletal	Long face, open bite of 1.5 mm, overjet of 1.93 mm;	Le Fort I: maxillary advancement 4.6 mm, intrusion of 1 mm BSSO: mandibular advancement 1.6 mm
P-10	22	M	Class III dentoskeletal	Long face, right mandibular deviation, open bite 4.1 mm, overjet – 5.2 mm	Le Fort I: clockwise 1 ° BSSO: mandibular intrusion 2.4 mm, advancement − 2.9 mm;

### TeethanTM sEMG results

A total of ten patients who underwent orthognathic surgery participated in this study. sEMG recordings were taken using TeethanTM at three time points: pre-operatively, at one month and at six months post-operatively. The sEMG data were collected from the masticatory muscles and reflected the occlusal contacts in the different clinical condition.

The data were analysed to identify significant changes in muscle activity: mainly, the difference in values recorded at times T0, T1 and T2 was considered and especially the values of POC, both temporal and masseter, and barycentre.

The evaluation of symmetrical muscle activity is a crucial aspect in assessing the balance and functionality of the masticatory system. Both temporal and masseter muscles play a significant role in chewing and jaw movement, and their activity patterns can provide valuable insights into the functional symmetry of these muscles. The choice to analyze the POC (Percentage Overlap Coefficient) of the temporal and masseter muscles allows for a detailed understanding of whether the muscles on both sides of the face are working harmoniously. Any asymmetry could indicate dysfunction or imbalance in stomatognathic system.

Additionally, the barycentre—or the centre of force distribution—serves as an important metric in evaluating occlusal equilibrium. Occlusal equilibrium refers to the proper alignment and distribution of bite forces across the dental arches during closure and functional movements. By analyzing the barycentre, clinicians can gain information about how well the occlusal forces are balanced and whether any discrepancies might be contributing to uneven force distribution or strain on specific teeth, muscles, or joints. Such an analysis is particularly valuable in both diagnostic assessments and in monitoring post-treatment outcomes to ensure optimal functional balance.

Other outcome parameters were not included in the analysis due to their substantial variability, which is largely influenced by the type of malocclusion present at baseline (e.g., IMPACT or TORS).

A descriptive statistical analysis was performed to evaluate the magnitude and variability of occlusal parameter changes across the observation intervals. For each parameter (POC t, POC m, and BAR), mean values and standard deviations (SD) were calculated for the overall sample and for two predefined subgroups of patients : group A, including patients with Class III malocclusion, and group B, including patients with Class II malocclusion. All analyses were conducted using absolute numerical values, disregarding directional indicators (R/L, A/P), as the aim was to quantify the extent of variation rather than its vectorial orientation. The parameters considered included the changes between T0–T1 and T1–T2 for POC t, POC m and BAR. No inferential statistics were applied due to the limited sample size and the exploratory nature of the study.

Considering parameters of total of patients, POC temporal average Δ T0 – T1 was 6.76 ± 8.36 and average Δ T1 – T2 was 5.68 ± 5.33; POC masseter average Δ T0 – T1 was 8.74 ± 7.38 and average Δ T1 – T2 was 8.12 ± 7.42; the mean value ΔT0 – T1 of barycentre was 7.36 ± 4.13 and mean ΔT1 – T2 barycentre value was 10.65 ± 9.17.

In evaluation of patients according to their dentoskeletal deformities, average Δ T0 – T1 and Δ T1 – T2 was evaluated separately in Class II and Class III dentoskeletal deformities: in Group A, POC temporal average Δ T0 – T1 was found to be 5.48 ± 6.31 and average Δ T1 – T2 was 5.98 ± 6.36; POC masseter average Δ T0 – T1 was 11.35 ± 8.63 and average Δ T1 – T2 was 11.05 ± 8.33; barycentre average Δ T0 – T1 was 7.08 ± 4.52 and mean Δ T1 – T2 barycentre value was 9.50 ± 7.53.

In Group B, POC temporal average Δ T0 – T1 was found to be 8.69 ± 13.31 and average Δ T1 – T2 was 5.23 ± 4.03, POC masseter average Δ T0 – T1 was 4.81 ± 3.11 and average Δ T1 – T2 was 3.72 ± 1.97, barycentre average Δ T0 – T1 was 7.80 ± 3.83 and barycentre average Δ T1 – T2 was 12.36 ± 13.15.

Changes in the electromyographic patterns of the ten patients were resumed in Table 3.

**Table 3:** Evaluation of difference of main sEMG parameters in time interval T0-T1 and T1-T2

Case	ΔT0 – T1	ΔT1 – T2
P-1	POC t: 0.03 L➔R	POC t: 2.71
	POC m: 0.88 L➔R	POC m: 8.25
	BAR: 1.68 A	BAR: 2.15 A ➔P
P-2	POC t: 4.34 R ➔ L	POC t: 2.48 L ➔ R
	POC m: 0.88	POC m: 3.79
	BAR: 9.8 P◊➔A	BAR: 9.17 A➔P
P-3	POC t: 17.81	POC t: 17.68%
	POC m: 24.67	POC m: 22.52 L➔R
	BAR: 10.52 A➔P	BAR: 4.94 P
P-4	POC t: 3.22 R➔L	POC t: 4.09 L➔R
	POC m: 6.1 L➔R	POC m: 0.82
	BAR: 1.94 P➔A	BAR: 1.97 A
P-5	POC t: 4.55	POC t: 1.27
	POC m: 9.32 R➔L	POC m: 20.01 L ➔R
	BAR: 3.23 P	BAR: 14.52 P
P-6	POC t: 0.23 R➔L	POC t: 0.23 L ➔R
	POC m: 6.46	POC m: 0.97
	BAR: 8.25 P	BAR: 22.7 P
P-7	POC t: 8.96	POC t: 9.45 L ➔R
	POC m: 6.76	POC m: 5.21
	BAR: 4.26 A➔P	BAR: 9.48 P
P-8	POC t: 26.08	POC t:3.07 R➔L
	POC m: 8.33 R➔L	POC m: 5.59
	BAR: 10.1 P	BAR: 6.03 P
P-9	POC t: 1.11	POC t: 11.28 L ➔R
	POC m: 3.94 L ➔R	POC m: 4.69
	BAR: 9.34 A	BAR: 32.27 A➔P
P-10	POC t: 1.27	POC t: 4.55
	POC m: 20.01 R➔L	POC m: 9.32
	BAR: 14.52 P	BAR: 3.23 P

### Evolution of TMD symptoms and correlation with sEMG findings

Among the ten patients included in the study, none exhibited clinical signs of temporomandibular joint damage or disc displacement. Four patients reported mild preoperative muscular pain (2 with Class II malocclusion and 2 with Class III malocclusion), localized to the masseter or temporalis muscles. In these cases, sEMG recordings at T0 revealed elevated asymmetry index values (ASIM > ± 10) and reduced IMPACT scores (< 100%), consistent with muscular imbalance and proprioceptive inhibition. At six months postoperatively (T2), three patients showed normalization of sEMG parameters with complete symptom resolution, while one patient reported mild residual discomfort. Results were resumed in Table 4.

**Table 4:** Summary of muscular symptoms and sEMG evolution

Patient (Type of malocclusion)	Symptoms	ASIM at T0	IMPACT at T0	ASIM at T1	IMPACT at T1	ASIM al T2	ASIM at T2	Symptoms evolution
P-1 (Class III)	Functional asymmetry, muscle overload, mild/severe pain of temporal/masseter muscles	−3.4	89.87%	+ 15.26	92.25%	+ 6.47	90.75%	Mild discomfort in masseter muscles
P-2 (Class II)	Mild masseter pain	−3.05	71.11%	−11.06	53.17%	+ 1.75	103.45%	Resolved
P-5 (Class III)	Mild temporalis pain	–14	89%	−3.2	101%	−6	104%	Resolved
P-9 (Class II)	Mild bilateral temporalis and masseter discomfort	+ 11	94%	+ 9.85	90%	+ 2	110%	Resolved
Others	None	Within range	Normal	Within range	Normal	Within range	Normal	No symptoms

These findings suggest that skeletal base repositioning and occlusal rebalancing may contribute to improved neuromuscular coordination and symptom relief, even in the absence of structural joint pathology.

## Discussion

Orthognathic surgery is a common procedure performed to correct various skeletal and dental deformities of the jaws [15, 16]. Currently, the movements of the jaws in the three dimensions of space are virtually programmed using various software, making it possible to predict the final result [17]. These movements not only improve the patient’s aesthetics but also directly affect the associated muscle vectors. Specifically, surgically induced skeletal modifications can alter electromyographic patterns, leading to changes in muscle contraction and force distribution during mastication [18]. The post-operative period is challenging for the patient in view of the major changes brought about by the surgical correction [19, 20]. In the literature, many indications are recorded for monitoring and evaluating these changes in such a sensitive period for the patient [21]. This phenomenon underscores the importance of thorough post-operative neuromuscular behaviour evaluation to better understand the functional adaptation process and optimize rehabilitative outcomes. In fact, as reported by Hellmann et al. [16], inputs from periodontal receptors play a crucial role in the precise motor control required for intra-oral manipulation, as well as for activities such as biting and chewing. As also, as outlined by Giraudeau, occlusal proprioception could affect also static posture [22].

Our observational study allowed us to analyse the variations in electromyographic patterns in patients undergoing orthognathic surgery, observing how masticatory vectors change in response to surgically induced skeletal alterations. This approach represents a first step towards a deeper understanding of the role of masticatory muscles in the context of dentoskeletal deformities and their management.

In the literature, there is conflicting data regarding the relationship between orthognathic surgery and joint disorders. Corrective orthognathic surgery appears to provide secondary benefit of treating TMD [23], even if different studies highlighted changes in condylar position, these did not cause clearly TMD [24].

According to one of the most recent systematic reviews, orthognathic surgery seems to have a beneficial effect on the signs and symptoms of temporomandibular disorders (TMDs). However, it remains challenging to predict the impact of the procedure on the positioning of the temporomandibular joint (TMJ) disc [25].

Moreover, the existing literature highlights a significant gap in research concerning the use of electromyography to monitor patients who have undergone orthognathic surgery for the potential development or progression of TMDs. Expanding investigations in this area could provide valuable insights into early detection and management strategies for muscle activity and occlusal dysfunctions in these patients.

In relation to patients with dental-skeletal deformities, sEMG could have a role to detect some parameters that are repeated in patients with malocclusion and that describe the patterns of increased activation and functionality of the masticatory muscles based on occlusal contacts. This balance and its evolution could be monitored in the pre- and post-surgical correction period to assess how the shift of the skeletal bases and the achievement of a normal dental occlusion can influence static muscle work [21, 26, 27].

Specifically, in Class II dentoskeletal, characterized by a retruded position of mandible, the TeethanTM device had provided detailed sEMG data:

- Barycentre (BAR) Assessment: the occlusal barycentre was posteriorized, indicating a backward shift in the occlusal balance point, because of major occlusal contacts in posterior sectors;
- Muscle Activation: increased activity in the masseter muscles was observed as these muscles, due to posterior occlusal contacts;
- Asymmetry (ASIM): elevated asymmetry index due to compensatory muscle activity on one side, in case of lateralization.

In Class III malformations, characterized by a protruded position of mandible, the Teethan^®^ device had offered insights:

- Barycentre (BAR) Assessment: the occlusal barycentre was anteriorized, due to greater occlusal contact between the anterior sectors and thus greater activation of the temporal muscles;
- Muscle Activation: increased activity in the temporal muscles was observed as these muscles work harder to achieve proper occlusal contact.
- Asymmetry (ASIM): elevated asymmetry index due to differences in muscle activity between the left and right sides.

In our cases, P-1, shown in Case 1, had a Class III dentoskeletal malocclusion with mandibular latero-deviation to the right and reduced occlusal contacts on the right side. At the initial evaluation (T0), the patient exhibited muscular balance based on their basic anatomy, with an electromyographic pattern showing a predominance of left masseter POC at 88.3%. This reflected the presence of more occlusal contacts on the left side, where the masseter muscle was more active. Following the repositioning of the skeletal bases and mandibular shift, with the centring of the midline, occlusal contacts were restored bilaterally, including those on the right side. This change led to an increase in right masseter POC, with a more balanced distribution of masticatory forces. Additionally, a significant variation in muscle activity symmetry was observed. Before surgery, the muscle component was greater on the left side due to predominant occlusal contacts in that area. Clinically, the patient presented a mild/severe pain at palpation of both temporal and masseter muscles, majorly represented in left masseter. However, after the skeletal bases shift, a new asymmetry was recorded, with a high percentage of muscle activity on the right side, where occlusal contacts were restored. This change in asymmetry suggests that the new muscular balance, consequent to improved occlusal contacts, led to a readjustment of neuromuscular coordination, with a more uniform force distribution between the two sides, even if the clinical pain of masseter muscles wasn’t disappear, unfortunately.

The P-2, shown in Case 2, had a Class II malocclusion with overjet and deep bite. At the initial evaluation (T0), there were no occlusal contacts in the anterior sectors. This resulted in an abnormal distribution of muscle forces, with a completely posterior barycentre, reflected in the EMG pattern. This situation indicated a misalignment between masticatory muscles and occlusal contacts, compromising the normal neuro-muscular balance. The patient referred mild pain in masseter muscle. After the surgery and the restoration of occlusal contacts at the level of the anterior and lateral incisors, a shift in the muscle barycentre was observed, moving it to an anterior position. This shift reflected the new balance created by the restored occlusal contacts and the readjustment of masticatory function, accompanied by disappearance of muscular pain. This phenomenon is easily understood, as once the occlusal change occurred, neuromuscular adaptation was necessary to respond to the newly established occlusal contacts.

As demonstrated by the difference in parameters reported in Table 3, higher values are obtained in patients who underwent surgical corrections and major mandibular shifts or with greater latero-deviation, reflecting a reactive muscular response to surgical correction.

It is evident that in the first post-operative month there is muscular imbalance and a significant flow of changes and muscle readjustments based on the new occlusal stimuli, as demonstrated in ΔT1-T0. Only in the T2 evaluation, six months after surgery, is it noted that the patient re-establishes their muscular and occlusal balance. Occlusal contacts that determine proprioceptive input are thus re-established, and the muscle adapts to the new anatomy and thus to the new occlusal component [28].

The descriptive statistical approach adopted in this study allowed us to quantify the magnitude and variability of these parameters in the overall sample and in two malocclusion-based subgroups. POC t, POC m and BAR indices provide complementary information on the bilateral coordination of the masticatory muscles and on the spatial distribution of occlusal load during mandibular closure.

Across the entire cohort, all parameters exhibited substantial variability, confirming the heterogeneity typically observed in electromyographic assessments of occlusal function. This variability reflects the influence of individual occlusal morphology, neuromuscular compensation strategies, and the intrinsic adaptability of the stomatognathic system. The relatively high standard deviations observed for both POC and BAR underscore the complexity of interpreting functional occlusal indices in mixed malocclusion populations.

Interestingly, POC t and POC m tended to stabilize during the second interval in both groups, whereas BAR remained more variable, particularly in Class II subjects. This divergence highlights the fact that muscular symmetry and occlusal load distribution may follow different adaptive pathways depending on the underlying skeletal and dental morphology.

Importantly, although no patients presented with structural temporomandibular joint (TMJ) damage or disc displacement, four individuals (Patients 1, 2, 5, and 9) exhibited muscular symptoms compatible with myogenic TMD, such as localized discomfort or tension in the masseter or temporalis muscles. In these cases, sEMG recordings at T0 showed elevated ASIM values and reduced IMPACT scores, consistent with neuromuscular imbalance and proprioceptive inhibition. Postoperative follow-up demonstrated normalization of these parameters in three patients, with complete symptom resolution. Patient 1, however, continued to report mild discomfort during prolonged mastication, despite improved sEMG indices, suggesting residual muscular sensitivity.

These findings support the hypothesis that orthognathic surgery, by reestablishing occlusal symmetry and skeletal alignment, may contribute to the alleviation of muscular TMD symptoms in selected cases. The dynamic evolution of ASIM and IMPACT values underscores the importance of monitoring not only occlusal outcomes but also functional muscle behaviour during postoperative recovery.

The purpose of the study is only descriptive and given the limited number of cases it is not possible to give a clear interpretation of the values obtained.

One of the central aspects that emerged is the role of sEMG in identifying and monitoring changes in electromyographic patterns. In particular, the ability to objectify variations in muscle contraction provides crucial information on neuro-muscular adaptation and post-surgical functional stability [29], making surface electromyography (sEMG) a promising therapeutic protocol.

While the findings of this pilot study offer promising insights into the neuromuscular adaptations following orthognathic surgery, it presents several limitations: first, the small sample size and the exploratory nature of the design did not allow for formal inferential statistical testing. For this reason, results were presented exclusively as descriptive measures and no conclusions regarding statistical significance can be drawn. The high variability observed in several parameters further underscores the heterogeneity of the sample and limits the generalizability of the findings. The wide dispersion observed in some parameters reflects the intrinsic biological variability of sEMG-based occlusal measurements, which are strongly influenced by individual occlusal morphology, neuromuscular compensation strategies, and the adaptive behaviour of the stomatognathic system. Second, although surface electromyography (sEMG) is widely used to assess neuromuscular function, independent validation studies specifically evaluating the Teethan™ device remain limited in the current literature. Third, no primary outcome was pre-specified, giving the descriptive nature of the study. More definitive outcomes may be established in a larger patient cohort; therefore, the present study has an exclusively descriptive aim.

Nevertheless, this study provides a valuable foundation for future investigations. Larger, multicentric studies with stratified patient cohorts and longer follow-up periods are needed to validate these preliminary observations and to explore the predictive value of sEMG parameters in post-surgical rehabilitation.

Future research should also consider dynamic EMG assessments, correlations with imaging modalities (e.g., CBCT, MRI), and the inclusion of patient-reported outcome measures to better capture the functional and quality-of-life implications of orthognathic interventions.

## Conclusions

This pilot study highlights the functional impact of orthognathic surgery on masticatory muscle activity, showing that skeletal repositioning leads to measurable neuromuscular changes detectable via surface electromyography (sEMG). In patients with dentoskeletal deformities, sEMG revealed early postoperative imbalance followed by progressive normalization, with notable improvements in symmetry and bite force. This study define adaptation patterns differing according to the underlying malocclusion type.

Although no joint damage was present, several patients showed muscular TMD symptoms preoperatively, which largely resolved after surgery. These findings underscore the value of integrating sEMG into orthognathic protocols to monitor functional recovery.

Given the limited sample size and the exploratory nature of the study, conclusions remain preliminary. However, this study establishes a foundation for larger investigations and could serve as a potential basis for exploring the use of sEMG as a diagnostic and follow-up tool in the field of orthognathic surgery.

## Data Availability

All data are reported in the manuscript.
